# Assessing the psychometric performance of EQ-5D-5L in dementia: a systematic review

**DOI:** 10.1186/s12955-022-02036-3

**Published:** 2022-09-28

**Authors:** Anju D. Keetharuth, Hannah Hussain, Donna Rowen, Allan Wailoo

**Affiliations:** School of Health and Related Research, University of Sheffield, Sheffield, S14DA UK

**Keywords:** EQ-5D-5L, Dementia, Psychometrics, Systematic review

## Abstract

**Background:**

EQ-5D is widely used for valuing changes in quality of life for economic evaluation of interventions for people with dementia. There are concerns about EQ-5D-3L in terms of content validity, poor inter-rater agreement and reliability in the presence of cognitive impairment, but there is also evidence to support its use with this population. An evidence gap remains regarding the psychometric properties of EQ-5D-5L.

**Objectives:**

To report psychometric evidence around EQ-5D-5L in people with dementia.

**Methods:**

A systematic review identified primary studies reporting psychometric properties of EQ-5D-5L in people with dementia. Searches were completed up to November 2020. Study selection, data extraction and quality assessment were undertaken independently by at least 2 researchers.

**Results:**

Evidence was extracted from 20 articles from 14 unique studies covering a range of dementia severity. Evidence of known group validity from 5 of 7 studies indicated that EQ-5D-5L distinguishes severity of disease measured by cognitive impairment, depression, level of dependence and pain. Convergent validity (9 studies) showed statistically significant correlations of weak and moderate strengths, between EQ-5D-5L scores and scores on other key measures. Statistically significant change was observed in only one of 6 papers that allowed this property to be examined. All seven studies showed a lack of inter-rater reliability between self and proxy reports with the former reporting higher EQ-5D-5L scores than those provided by proxies. Five of ten studies found EQ-5D-5L to be acceptable, assessed by whether the measure could be completed by the PwD and/or by the amount of missing data. As dementia severity increased, the feasibility of self-completing EQ-5D-5L decreased. Three papers reported on ceiling effects, two found some evidence in support of ceiling effects, and one did not.

**Conclusions:**

EQ-5D-5L seems to capture the health of people with dementia on the basis of known-group validity and convergent validity, but evidence is inconclusive regarding the responsiveness of EQ-5D-5L. As disease progresses, the ability to self-complete EQ-5D-5L is diminished.

**Supplementary Information:**

The online version contains supplementary material available at 10.1186/s12955-022-02036-3.

## Background

With an increasing incidence of people living with dementia (PwD), the number of studies investigating novel interventions and strategies for the management and care of dementia is on the rise [1], which in turn, may lead to increased pressure on the limited resources of the NHS. Having the right outcome measures to adequately capture the benefits of treatments for this population is essential to ensure the efficient allocation of resources. Concerns around the challenges posed by issues of cognition, time perception, memory and judgement have questioned the suitability of existing preference-based measures (PBMs) to compute quality adjusted life years (QALYs) in PwD [2].

In the UK, the EQ-5D is the preferred measure of health-related quality of life (HRQoL) by the National Institute of Health and Care excellence (NICE) to generate QALYs for use in economic evaluation [3]. The descriptive system comprises five dimensions reflecting generic HRQoL: mobility, self-care, usual activities, pain and discomfort and anxiety and depression [4]. In addition to the descriptive system, EQ-5D has preference weights from several countries allowing health state utility values to be estimated that reflect the societal preferences of the given country, which can be integrated into country-specific economic evaluations. There are two versions of the EQ-5D, the EQ-5D-3L [4] and the EQ-5D-5L [5]. The 3L version has three response levels of severity for each of the five dimensions and the 5L version was later introduced to improve the instrument’s sensitivity and reduce ceiling effects by increasing the number of severity levels [5]. It has the same five dimensions, with two additional levels of severity. The EQ-5D can be self-completed or administered by interviewer, and in particular cases can be completed via a proxy assessor—which describes when a person is asked to report on behalf of someone else in relation to their health status. The proxy should be someone that knows the patient well for example, a family member or friend, caregiver or healthcare professional [6].

A recent systematic review of utility measures for PwD, based on 64 published studies, found that EQ-5D-3L was the most widely used measure in cost-effectiveness analyses (34 studies) [7]. The other measures used were: Dementia Quality of Life (DEMQOL)-U (utility score) [8] (n = 2), Health-Utility Index (HUI) [9] (n = 17), Quality of Wellbeing (QWB) [10] (n = 4), Assessment of Quality of Life (AQoL-8D) [11] (n = 2) and 15-D (n = 3). EQ-5D-3L was considered the most feasible and acceptable in terms of completion time, response rate and the number of missing items. In terms of precision, ceiling effects have been observed for EQ-5D-3L and other measures. The majority of evidence pertained to the three-level version of EQ-5D and there is a lack of evidence on the more recent five-level version, EQ-5D-5L.

Concerns have been raised around the content validity of PBMs to reflect the themes that are important for PwD. QWB was found to have the highest number of relevant items [7]. A more recent study assessing the face and content validity of six preference-based measures suggested that participants did not express a clear preference for one over the other [12]. When responsiveness was assessed, only EQ-5D-3L was found to have an effect size greater than 0.5, underscoring the need for more evidence on this property. In summary, EQ-5D-3L remained the most widely used PBM mainly by virtue of its brevity. The majority of the evidence on EQ-5D in this population uses the 3L version. While in theory, the EQ-5D-5L may be more sensitive and less subject to ceiling effects, the five responses may pose extra challenges for PwD. A recent systematic review of the psychometric performance across conditions found that the EQ-5D-5L exhibited excellent psychometric performance, but this did not fully assess the evidence on EQ-5D-5L usage in dementia [13].

The purpose of this paper was to assess the psychometric performance of EQ-5D-5L in a population of PwD with a view to help inform the suitability of the measure for generating utilities and QALYs to inform economic evaluation. The objectives were to identify published literature on the psychometric properties of EQ-5D-5L in PwD and conduct a systematic review of the published literature.

## Methods

EQ-5D-5L has five dimensions: mobility, self-care, usual care, pain/discomfort, anxiety/depression. Each dimension has five levels: no problems, slight problems, moderate problems, severe problems and extreme problems.

### Literature searches

A systematic search was conducted in Medline (Ovid), the Web of Science Core Collection Science Citation Index Expanded (Clarivate Analytics) and PsycINFO from 2009 (date when EQ-5D-5L became available) to Nov 2020 to identify studies reporting the psychometric performance of EQ-5D-5L in PwD. Search terms for the measures and the population are shown in Table 1. The search strategy was translated across each database and limits for human studies and English language were applied. No study type limit was applied. Supplementary grey literature searches included the conference abstract websites in the last three years from the International Society for Pharmacoeconomics and Outcomes Research and International Society for Quality of Life Research, Web of Science Cited Reference Search, keyword searching using Google Scholar search engine and examination of reference lists of included studies.

**Table 1:** Final MEDLINE strategy

**#**	Searches
1	(dementia or Alzheimer*).mp
2	(euro qual or euro qual5d* or euro qol5d* or eq-5d* or Eq. 5-d* or Eq. 5d* or euroqual or euroqol or euroqual5d* or euroqol5d*).ti,ab,kf
3	1 and 2
4	limit 3 to (english language and yr = "2009 -Current")

### Study selection

Eligible papers (full-text articles and abstracts without available free full versions online) were selected by two reviewers (AK and HH). Eligibility criteria are summarised in Table 2. After excluding duplicates, titles and abstracts, all potentially relevant articles were obtained for detailed review. Disagreements were resolved by discussion with a third reviewer present (DR).

**Table 2:** Study eligibility criteria

	Inclusion criteria	Exclusion criteria	Additional Notes relating to study eligibility
Population	People with dementia	People without dementia	We have included papers with an elderly population where the results were reported separately for people with dementia
Outcome	EQ-5D-5L	Not EQ-5D-5L	We included papers where psychometric information could be extracted even though the purpose of the study was not a psychometric study per se
		EQ-VAS only	
Study design	Any design	N/A	
Language of published article	English	Non-English	Studies using non-English versions of the measure were included

### Data extraction

Three reviewers (HH, AK, DR) independently extracted psychometric evidence for the same three very different papers purposefully selected [14–16], compared their findings and resolved any disagreement to ensure a standard approach to data extraction for the remaining papers. Thereafter, each of the two reviewers (DR, HH) extracted half of the remaining papers and a final check was carried out by a third reviewer (AK).

Data extraction for this review was performed using similar methods to a previous review [17]. Data on the following were extracted: study aim; country; language of the EQ-5D-5L; mode of administration; preference weights to generate EQ-5D-5L scores if used; age range of participants; mean age; gender proportions; sample size; other measures; disease and severity reported; whether the measures have been self-reported or proxy-completed; whether the analysis uses scores, dimensions or both and the other measures reported. Data assessing the psychometric properties of known-group validity, convergent validity, responsiveness, reliability and acceptability described below were also extracted. Known-group validity measures whether the instrument is able to differentiate between different groups with different severity. To do so, a measure of severity is needed as well as hypotheses to be tested, for example, people with more severe impairment are likely to have lower quality of life, and we have used the a priori hypotheses identified by the authors (either explicitly or implicitly) of each study. Known-group validity is indicated if a statistically significance difference at the 5% level across known groups is observed, along with whether the direction of the difference is in accordance with clinical expectation. Known-group difference can be measured by standardised effect sizes (ES) often dividing the mean by the standard deviation of the milder group where ES of 0.2 is normally considered small, 0.5 moderate, and 0.8 large [18]. Convergent validity measures the degree of association between the measure of interest (EQ-5D-5L) and other health-related quality of life measures, and this can be at item/dimension level or using sum scores of scores where appropriate. Convergent validity is more often assessed using correlation coefficients but can also be assessed using statistical significance from regression analyses. In this review, a correlation coefficient of ≥ 0·70 is taken as strong evidence of construct validity with the additional categories: ≤ 0.40—weak correlation and moderate correlation lies between 0.41 and 0.70 [19]. Evidence of convergent validity focuses upon expected correlations motivated in theory. Test–retest reliability assesses the ability of the measure to produce consistent values in cases where no changes in health-related quality of life is expected. Inter-rater reliability refers to the ability of different raters completing the measures to produce consistent values. Intra-class coefficients are often used to measure test–retest reliability. Responsiveness is the ability to reflect change over time in cases where change is expected, for example following treatments. Evidence of responsiveness is present if a statistically significance change at the 5% level over time is observed. The direction of the change is also considered to determine whether it is in accordance with clinical expectation e.g. higher HRQoL post-treatment compared to baseline. Acceptability and feasibility refer to the practicality of administering a measure and the ease with which it is completed by the patients. They cover aspects such as time taken to complete the measure, whether assistance is needed and missing data, the latter being an indication of the ease with which the measures can be completed. A lack of evidence for acceptability and feasibility is concluded where the study reports, for example, high levels of missing data or low levels of understanding. We have reported ceiling effects separately as it is an important consideration given the context of EQ-5D-5L. Ceiling effects are said to be present when there are significant number of respondents score the highest possible value. Amongst the different cut-offs in the literature, in this review we have taken the cut-off to be 15% [20] as this is also stated by one of the papers [21].

### Quality assessment

This review allowed for the inclusion of all study types (clinical studies, cost-effectiveness analyses, observational studies etc.). Therefore, rather than using pre-existing quality appraisal tools (which tend to be targeted to a specific study-type), the standardised GRADE assessment tool was adapted and used to perform a less formal quality appraisal of the papers [22]. The assessment criteria comprised 11 questions around the population, study sample size and outcome administration methods used within the study, whether details of analysis were provided, quality of data and whether selection bias was discussed. Each question was scored and the total score was used to categorise papers are high, medium and low (details in Additional file1).

## Results

**Fig. 1:**
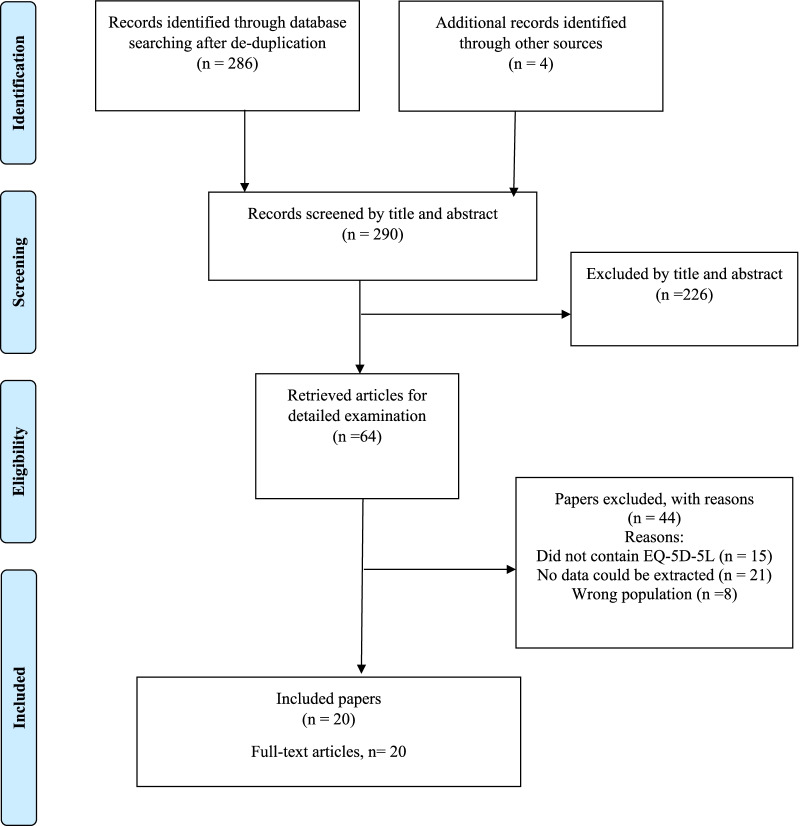
PRISMA diagram outlying flow of study selection

Of the 511 records retrieved from the three databases searches, 225 duplicates were removed, and 20 studies were found to be eligible for inclusion in the review. Forty-four studies were excluded because they did not include EQ-5D-5L, were from the wrong population or no meaningful psychometric data could be extracted (Fig. 1).

## Summary of included studies

The 20 papers in this review related to 14 unique studies: with four papers from the Access to Timely Formal Care Cohort (Actifcare) study [23–26], and three from the Enhancing person Centred Care in Care Homes (EPIC) trial [27–29], and two from the INSPIRED study [14, 16] (Table 3). The studies were carried out in a number of countries with the highest number of papers from the UK (n = 7) from 5 different studies and Australia (n = 5) from four different studies, four countries with one paper each (Denmark, Italy, Japan and Singapore) and four papers from one multinational study (Germany, Ireland, Italy, the Netherlands, Norway, Portugal, Sweden and United Kingdom).

**Table 3:** Characteristics of included studies (20 studies)

Study references	Country	Study aim/type	Index or dimensions or both	Preference weights	Setting	Health Condition as reported by author	Self-report	Proxy report, details	Mean age (SD)	% female	N
Easton, 2018 [14]	Australia	Psychometric analysis (trial data)	Both	England (Devlin et al. 2016)	Residential care homes (n = 17) across 4 states	Cognitive impairment, dementia and disability	Yes	Yes	85.5 (8.5)	74.5	541
Engel, 2020 [12]	Australia	Qualitative (content and face validity)	Dimensions	Not applicable	Community dwelling	Mild dementia and carers of people with dementia	Not applicable	Not applicable	74.9	44.0	26
Griffiths, 2020 [27]	UK	Psychometric analysis (trial data)	Both	England (Devlin et al. 2016)	Residential care homes (n = 50)	Formal diagnosis of dementia (or > 4 on the FAST)	Yes	Yes	85.6 (7.6)	73.8	726
Handels, 2018 [23]	8 European countries	Cross-sectional cohort study	Index	van Hout crosswalk UK (2012)	Community dwelling	Mild to moderate dementia	Yes	Yes	78.0 (8.0)	55.0	451 dyads
Harrison, 2018 [16]	Australia	Cross-sectional cohort study	Index	England (Devlin et al. 2016)	Residential care homes (n = 17) across 4 states	Older adults 64.3% of whom had a dementia diagnosis	Yes	Yes	85.5 (8.5)	74.5	541
Hurley, 2020 [42]	UK	Feasibility study	Neither	Not applicable	Residential care homes (n = 3)	Care home residents (majority with mild dementia)	Yes	No	89.0	82.9	35
Janssen, 2018 [24]	8 European countries	Cross-sectional cohort study	Both	England (Devlin et al. 2016)	Community dwelling	Mild to moderate dementia	No	Yes	77.4 (7.8)	53.0	390 dyads
Jurkeviciute, 2019 [41]	Italy	Business case development	Both	Spain (no reference provided)	Community dwelling	Elderly with Mild Cognitive Impairment and Mild Dementia	Not reported	Not reported	79.0 (6.0)	57.4	107
Maidment, 2020 [31]	UK	Feasibility study	Index	van Hout crosswalk UK (2012)	Residential care homes (n = 5)	Moderate to severe dementia	No	Yes	83.6 (9.3)	62.1	29
Martin, 2019 [28]	UK	Psychometric analysis (trial data)	Index	England (Devlin et al. 2016)	Residential care homes (n = 50)	Dementia	Yes	Yes	85.5	73.2	1004
Meads, 2020 [29]	UK	Cost-effectiveness study	Index	England (Devlin et al. 2016) van Hout crosswalk UK (2012)	Residential care homes (n = 50)	Dementia	Yes	Yes	85.6 (7.6)	74.0	726
Perry-Duxbury, 2020 [25]	8 European countries	Psychometric analysis	Index	England (Devlin et al. 2016)	Community dwelling	Mild to moderate dementia	No	Yes	77.7	54.5	451
Ratcliffe, 2017 [36]	Australia	Psychometric analysis (trial data)	Index	England (Devlin et al. 2016)	Nursing Care Facilities (n = 3)	Frail older adults, 92.5% of whom had moderate to severe dementia	Yes	Yes	88.6 (5.6)	74.2	240
Rombach, 2020 [26]	8 European countries	Statistical mapping study	Index	van Hout crosswalk UK (2012)	Memory clinics, general practices, community mental health teams	Dementia	Yes	Yes	78.0 (8.0)	55.0	451
Sopina, 2019 [34]	Australia	Empirical analysis of secondary data	Both	Australia (Viney et al. 2011)	Nursing homes (n = 20)	Advanced dementia (palliative stages of care)	No	Yes	85.0 (8.0)	63.0	284
Sopina, 2017 [35]	Denmark	Cost-effectiveness study	Index	Wittrup-Jensen KU et al. 2009 Danish TTO	Memory clinics	Mild Alzheimer's disease	Yes	Yes	70.0 (7.4)	43.0	200
Toh, 2020 [21]	Singapore	Feasibility/Psychometric analysis	Both	van Hout crosswalk (2012)Singapore	Nursing homes (n = 3)	Nursing home residents (% with dementia not reported)	No	Yes	73.4 (13.5)	51.3	229
Umegaki, 2020 [30]	Japan	Empirical analysis of primary data	Both	Not reported	Memory Clinic (n = 1)	Mild to moderate dementia	Yes	Yes	80.1 (5.4)	54.1	74
Usman, 2019 [40]	UK	Empirical analysis of secondary data	Both	van Hout crosswalk UK (2012)	Residential care homes (n = 24)	Dementia and cognitive impairment	Yes	Yes	86.8 (7.6)	68.0	117
van de Rijt, 2020 [37]	UK	Empirical analysis of primary data	Index	Not reported	Nursing homes (n = 4)	Dementia ( 63.1% were categorised as "severe")	Yes	No	83.9 (8.0)	62.2	111

There were several languages for EQ-5D-5L used in the papers: English (n = 13), Japanese (n = 1), Italian (n = 1), Danish (n = 1) and local languages for the multinational studies (n = 4). In the case of four papers, the language was not stated and had been assumed to be English [21] and Japanese [30].

The papers recruited participants in different settings: residential care homes (n = 8), community dwellings (n = 6), nursing homes (n = 4) and memory clinics (n = 2). While all the studies assessed patients with dementia, there was a wide range of severity where specified: mild dementia (n = 3), mild to moderate (n = 4), moderate to severe (n = 2), advanced (n = 1) and mild Alzheimer disease (n = 1). One study among nursing home residents did not specify the percentage of participants with dementia but it was selected for inclusion because the authors stated that participants were selected through stratified sampling according to the resident’s dementia status and functional diagnosis [21].

Sample size varied considerably across studies ranging from 26 (qualitative study) [12] or 29 [31] to 1004 [28]. Three papers had sample sizes less than 50, one between 51 and 100, four between 101 and 200, seven between 201 and 500, four between 501 and 750 and one greater than 750.

Ten studies assessed the EQ-5D-5L index score only, one study only assessed the dimensions, eight included both dimensions and index score and one qualitative study did not explicitly consider either. Twelve of the 20 studies reported using UK specific preference weights with four using the cross-walk from EQ-5D-5L to EQ-5D-3L [32]; eight used values from the value set for England produced by Devlin et al. [33]; one used both sets mentioned; the value sets used by three papers was unclear though there is some reference to UK values. One paper used the Australian weights, one used a crosswalk from Singaporean 3L value set, one used the Spanish preference values and, the preference weights used was unclear in a further four papers. Sopina et al. clearly stated using EQ-5D-5L but the preference weights used of those elicited for EQ-5D-3L and it was not possible to infer exactly how the weights for EQ-5D-5L were generated [34, 35]. One paper analysed dimensions only and one qualitative paper did not consider any value sets.

### Known-group validity

We were able to assess known-group validity from information provided in seven papers. Five papers significantly captured known-group differences for PwD with different degrees of unmet needs, with different levels of physical function and communication ability, people with or without sarcopenia (condition with loss of muscle mass and function) and for people with and without dementia (Table 4). Known-group differences were not observed in one study assessing a ‘facilitated family case conferencing’ intervention (similar to care planning with a multidisciplinary team) [34]. Although one study found mixed evidence for self-report and proxy completed scores at two different time points, the overall direction pointed to the fact that EQ-5D-5L scores were able to distinguish between different severity levels as measured by cognitive impairment, depression, level of dependence (self-care) and pain level [36]. The majority of results found that the differences were in the direction expected. Easton et al. [14] investigated both dimensions and the index and while the results were in the direction expected when assessed by different levels of cognition and functional impairment, they found that those with a diagnosis of dementia had higher EQ-5D-5L scores that those without. Another paper found no difference between those with and without dementia [37].

**Table 4:** Known-group validity (7 studies)

Study references (author, year)	Index or dimensions or both assessed	Groups defined by	Significant differences	Effect size	Mean differences across groups in direction consistent with clinical expectation
			Yes/No		
Easton, 2018^a^ [14]	Both	Cognition impairment [PAS-Cog score]	Yes	Small	No. PwD (self-report) with more impairment have higher EQ-5D-5L score
		Functional impairment in terms of dependence [MBI score]	Yes	Small to moderate	Yes as impairment increases, EQ-5D-5L score decreases
		Dementia status: with a diagnosis vs. without a diagnosis	No	Small	No. People with dementia have higher EQ-5D-5L score
Handels, 2018^b^ [23]	Index	Unmet need [no unmet need, 1 or 2 unmet needs, and 3 or more unmet needs]	Yes	Small^c^	Yes. People with more unmet needs have lower EQ-5D-5L score
Ratcliffe, 2017^a^ [36]	Index	Cognitive impairment [MMSE]	Yes	Small	Yes. Those with more severe levels of cognitive impairment have lower EQ-5D-5L score
		Depression [CSDD score]	Yes	Small	Yes. Those with more severe depression have lower EQ-5D-5L score
		Self-care [MBI dependence score]	Yes	Small	Yes. Those with more impairment have lower EQ-5D-5L score
		Pain [PainAd score]	Yes	Small	Yes. As pain increases EQ-5D-5L score decreases
Sopina, 2019^b^ [34]	Both	Facilitated family case conferencing (similar to care planning) versus with usual care	No	Small	Yes. Those participants with exposure to the case conference had higher EQ-5D-5L scores
Toh, 2020^a^ [21]	Both	Physical function and communication ability [RAF—Resident Assessment Form]	Yes	Small	Yes. Those with less impairment had higher EQ-5D-5L scores
Umegaki, 2020^b^ [30]	Both	People with and without sarcopenia	Yes	Small	Yes. Those with sarcopenia had lower EQ-5D-5L scores
van de Rijt, 2020^b^ [37]	Index	People with and without dementia	No	Small	No difference between the two groups

*CSDD* The cornell scale for depression in dementia, *MBI* modified barthel index, *MMSE* mini-mental state examination, *PainAd* pain assessment in advanced dementia scale, *PAS-Cog* cognitive impairment scale of the psychogeriatric assessment scale, *RAF* resident assessment form

^a^Hypotheses were explicitly stated by authors

^b^Hypotheses were implicitly stated by authors

^c^As reported by authors—exact figures not provided

### Convergent validity

As shown in Table 5, nine studies assessed convergent validity, with all of them finding statistically significant correlations with the other measures included in the studies, which are measures commonly used in dementia. However, the strength of these associations was varied. While one study did not report the exact correlation coefficient [28], of the remaining eight studies, half reported weak associations [14, 27, 34] (r < 0.4) and the other half found moderate associations [21, 24–26] (r = 0.41–0.7), with none of the studies reporting strong evidence of convergent validity between the measures. All of the studies with weak (but significant) associations were analysing the relationship between EQ-5D-5L and dementia-specific QoL measures i.e., DEMQoL-U, DEMQoL-U-proxy, QoL-AD, Quality of Life in Alzheimer’s disease scale—Nursing Homes version (QOL-AD-NH) and Quality of life in late-stage dementia (QULAID) [38]. Two studies explored relationships with ICEpop CAPability measure for Older people (ICECAP-O) [39], and reported moderate (significant) associations with both self [24] and proxy reported [25] EQ-5D-5L.

**Table 5:** Convergent validity (9 studies)

Study references (author, year)	Other HRQoL measures examined for correlation	Significant correlations	Regression analysis undertaken	Regression analysis shows significant relationship yes/no
Easton, 2018 [14]	DEMQoL-U and DEMQoL-proxy-U	Yes—EQ-5D-5L and DEMQOL-U (r = 0.346); EQ-5D-5L utilities and DEMQOL-U (r = 0.389)	No	NA
Griffiths, 2020 [27]	QUALID, DEMQoL-proxy, QoL-AD nursing home	Yes—EQ-5D-5L self-report with QUALID staff (r = 0.11) and relative proxy (r = 0.33), QoL-AD self-report (r = 0.3), DEMQoLstaff (r = 0.12) and DEMQoL relative proxy (0.39)	No	NA
Janssen, 2018 [24]	ICECAP-O	Yes—positive significant correlation between ICECAP-O and EQ-5D-5L utilities at baseline (r = 0.47)	No	NA
Martin, 2019 [28]	DEMQoL-Proxy-U, QOL-AD-NH, QUALID	Yes—resident-reported EQ-5D-5L and formal-carer–completed QUALID (r rated as high but authors—exact figure not reported)	Yes	Yes
Perry-Duxbury, 2020 [25]	ICECAP-O in the informal caregiver	Yes—ICECAP-O tariff significantly associated with EQ-5D-5L utility tariff score (r = 0.46)	Yes	Yes
Ratcliffe, 2017 [36]	DEMQOL-Proxy U	Yes—Proxy completed EQ-5D-5L and DEMQOL-Proxy U; Yes—EQ-5D-5L and MMSE (r = 0.22 at baseline)	No	NA
Rombach, 2020 [26]	QoL-AD scores and EQ-5D-5L utilities. In Additional File1 also reported for QOL-AD items and EQ-5D-5L dimensions	Yes—between similar dimensions in QOL	Yes	Yes
		Yes—between self-rated QoL-AD and EQ-5D (r = 0.49); Proxy QoL-AD and proxy EQ-5D (0.48 for one dataset and 0.56 for another)		
Sopina, 2019 [34]	QUALID	Yes—significant correlations between QUALID and EQ-5D-5L (r lies between − 0.3 and − 0.437 at different time points)	Yes	Yes
Toh, 2020 [21]	Domains of EQ-5D-5L and DCM WIB	Yes—significant correlation between EQ-5D-5L index and the DCM Well/Ill being value (r = 0.433)	No	NA

*CDR* Clinical dementia rating, *DCM WIB* dementia care mapping Well/Ill being (score), *CDR* DEMQOL-Dementia Quality of Life, *DEMQOL-U* Dementia Quality of Life Utility measure, *FAST* Functional Assessment Screening Tool, *ICECAP-O* ICEpop CAPability measure for Older people, *QoL-AD* Quality of Life—Alzheimer Disease, *NA* not applicable, *QoL-AD NH* Quality of Life-Alzheimer Disease Nursing Home version, *QUALID* Quality of Life in late-stage dementia

The lowest correlations were found between EQ-5D-5L completed by the PwD and other dementia measures (e.g. QUALID) completed by staff proxies.

### Reliability

Seven studies assessed the inter-rater reliability of EQ-5D-5L comparing completion by PwD and other proxies: staff proxies only (n = 2); family members or friends or informal carers (n = 4); and one study included one of the proxies mentioned and one included all formal and informal proxies as well as staff (Table 6). There was clear evidence from all the studies of the lack of inter-rater reliability between self-report and other proxy raters. One study reported fair agreement between staff proxy and informal carer proxies [27] and stated that for EQ-5D-5L dimensions, residents rated themselves as having ‘no problems’ more frequently than either relative/ friend proxies or staff proxies. The difference was particularly large for self-care, where one study found that 76% of residents stated they had no problems whereas staff and relative/friend proxies rated a much lower percentage of people with no problems (14% and 10%, respectively) [27]. Usman et al. [40] reported fair agreement for the mobility dimension and lower agreement for the remaining EQ-5D-5L dimensions. Across the studies, the overall EQ-5D-5L scores reported by PwD were higher than the scores recorded by proxies. Martin et al. [28] stated that these differences were more pronounced at the low end of utilities, namely as severity increased.

**Table 6:** Reliability (7 studies)

Study references (author, year)	Index or dimensions or both assessed	Analysis	Reliability observed yes/no
Griffiths, 2020 [27]	Both	Inter-rater reliability by self, proxy (relatives or friends or care staff); weighted Cohen’s Kappa statistic	No
Handels, 2018 [23]	Index	Inter-rater reliability by self and proxy (informal caregiver); paired t-tests	No
Martin, 2019 [28]	Index	Inter-rater reliability by self, proxy (formal and informal carers) assessed by spearman rank-order correlation and Bland Altman plots	Overall No
Sopina, 2019 [34]	Both	Inter-rater reliability; self and proxy (nurse). Intra-class correlation coefficients for residential facilities and two-way mixed effects model regression	No
Sopina, 2017 [35]	Index	Inter-rater reliability; self and proxy (main caregiver); Probability of being cost-effective—sensitivity analysis	No
Umegaki, 2020 [30]	Both	Correlation analysis between self and proxy (main caregiver)	No
Usman, 2019 [40]	Both	Inter-rater reliability staff proxy and self-complete at three time points. Weighted kappa statistics and intra-class correlation coefficients (ICCs) adjusted for clustering at the care home level were used to measure agreement between resident and staff proxies for each time point	No

### Responsiveness

The results from six studies assessing responsiveness are presented in Table 7. For five of the studies, responsiveness was assessed in the context of an intervention and in one study [28, 29, 34, 35, 41], change was assessed in the post-hospitalisation following a hip fracture [36]. All studies assessed the EQ-5D-5L index over time from baseline to one or up to three follow-up points. Five of the studies found changes in the direction expected, but of these two did not find that the change was statistically significant and one did not report on statistical significance. One study reported significant change for EQ-5D-5L proxy-completed by staff and relatives but not when self-completed by the PwD. One study which collected follow-up responses to assess the feasibility of doing so was not included in the table as the authors did not perform any analysis given the small sample size (n = 9) [31].

**Table 7:** Responsiveness (6 studies)

Study references (author, year)	Index or dimensions or both assessed	Comparison e.g. change over time	Comparison in direction consistent with clinical/expected expectation	Responsiveness of measure is statistically significant
Jurkeviciute, 2019 [41]	Both	Change over time from baseline to 6 months follow-up	No	No
Martin 2019 [28]	Index	Change over time from baseline to 2 follow-up points (exact timings not specified)	Yes	No for self-report and Yes for EQ-5D-5L-proxy and the informal-carer EQ-5D-5L-proxy
Meads, 2020 [29]	Index	Change over time from baseline to 6 and 16 months follow-up	Yes	Not reported
Ratcliffe, 2017 [36]	Index	Change over time from baseline to 4 weeks follow-up	Yes	Yes
Sopina, 2019 [34]	Index	Change over time from baseline to 6, 9 and 12 months follow-up	Yes	No
Sopina, 2017 [35]	Index	Change from baseline to 16 weeks follow-up	Yes	No

### Acceptability and feasibility

Ten studies assessed acceptability and feasibility of EQ-5D-5L as presented in Table 8. Six papers used missing data, one of which also analysed ceiling/floors effects, one study assessed the ability to complete, one qualitative study assessed people’s opinion from interviews, and one paper did not specify the analysis performed but reported a conclusion. Five studies found EQ-5D-5L to be acceptable to PwD assessed by whether the measure could be completed by the PwD and/or by the amount of missing data. The percentage of missing data for EQ-5D-5L for the PwD, when reported, ranged between 1 and 77%. Easton et al. [14] concluded that self-completion was feasible for only part of the population. Similar findings were observed by three other papers [28, 29, 42]. The studies found that as severity increased, the feasibility of collecting EQ-5D-5L data from PwD decreased, for example Griffiths et al. [27] found that PwD were too tired, and some had severe cognitive impairment hence were unable to complete the measure.

**Table 8:** Acceptability and feasibility (10 studies)

Study references (author, year)	Analysis	% EQ-5D data missing	Acceptability and feasibility observed
Easton, 2018 [14]	Not reported	NA	Yes partly
Engel, 2020 [12]	Interviews	NA	Yes
Griffiths, 2020 [27]	Missing data	< 1% (PwD)	Yes
Handels, 2018 [23]	Missing data	< 8% (PwD)	Yes
Harrison, 2018 [16]	Proportion of people in the study not able to self-complete assessments therefore proxy was used	< 1% (for proxy)	No (only proxy was used)
Hurley, 2020 [42]	Ability to complete	NA	No for self-complete; Yes for proxy
Janssen, 2018 [24]	Missing data	Not clear	Yes for proxy
Martin 2019 [28]	Missing data	44% (PwD)	No for self-report
Meads, 2020 [29]	Missing data	77% (PwD)	No for self-report
Toh, 2020 [21]	The feasibility criteria for missing data and ceiling/floor effects were ≤ 5% and ≤ 15% respectively	2.6% (PwD)	Yes

*NA* not applicable

### Ceiling effects

Ceiling effects were assessed by three papers. As mentioned in Table 8, one paper did not find any ceiling effects associated with the use of EQ-5D-5L in PwD [21]. One paper found evidence of ceiling effects for both EQ-5D and DEMQOL-U [14] and a further paper stated that half of the respondents in their sample had full utility scores [30].

### Quality assessment

Out of the 20 papers, four were of high quality, 12 were medium, two low and a score could not be determined for the qualitative paper included in the review [12] (see Additional file1 for the quality assessment).

## Discussion

This review has assessed the psychometric evidence of EQ-5D-5L in PwD based on 20 papers from 14 unique studies. Participants were recruited from a number of settings (residential, community dwelling, nursing homes, memory clinics) at different stages of dementia (from mild to severe) and a wide range of sample sizes, all adding to the heterogeneity of the population and the studies. Only a small number of papers assessed the psychometric properties of interest: known-group difference (n = 7); convergent validity (n = 9); responsiveness (n = 6); reliability (n = 7); and acceptability and feasibility (n = 10). The findings indicated that EQ-5D-5L scores could distinguish between known-groups of different severities as measured by cognitive impairment, depression, level of dependence and pain. Evidence of weak to moderate convergent validity was found in all papers assessing it. The weakest associations were present between self-completed EQ-5D-5L and staff completed outcome measures, which may be expected due to the otherwise observed inter-rater relationships. Out of the six papers assessing responsiveness, four papers did not show any significant changes though all reported changes in the expected direction. There was clear evidence of the absence of inter-rater reliability between self and proxy reports. While there was some evidence to support acceptability and feasibility of self-report EQ-5D-5L across six papers out of ten examining this, concerns were raised about burden and severe cognitive impairment jeopardising the ability of PwD to self-complete the measure.

Nine of the papers presented results for the EQ-5D-5L index only and nine presented results for both EQ-5D-5L dimensions and the utility index. The value set used was extracted when it was reported. There are currently 29 published value sets available that were generated using the standardised valuation techniques and protocol recommended by the EuroQoL Group [43]. There is evidence in the literature that utilities and results of cost-utility analyses are dependent on value sets used [44, 45]. By extension, some psychometric properties can be influenced by the value set especially where the utility scores have been used to assess the property. In the UK, the valuation of the EQ-5D-5L using time trade-off is currently in progress. There is a previous England value set that used a hybrid time-trade-off (TTO) and discrete choice experiment approach [33]. Currently the National Institute for Health and Care Excellence (NICE) [3] recommends the published mapping function to obtain EQ-5D-5L utilities from the EQ-5D-3L value set [3, 46, 47]. Therefore, as new value sets become available and more papers published using them, the psychometric properties of the EQ-5D-5L may need to be reassessed.

The evidence assessed is limited due to several reasons. First, there is a limited number of studies (14 studies from 20 papers). From the initial search, we retrieved 64 full articles and excluded 44 because either they used EQ-5D-3L which was not evident from either the abstract or the title, or no psychometric properties could be extracted, or the study assesses another population. Second, the quality of reporting in several of the papers was not ideal for the assessment of psychometric properties. This was mainly because the aim of only seven papers in this review was to psychometrically assess the properties of measures, while the rest have broader aims, for example cost-effectiveness analyses or assessing pain in people with and without dementia. As a result, we did not use any guidelines often used to assess the methodological quality of the studies. Third, we found limited evidence on content validity and this is an important psychometric property.

In assessing the evidence, a lot of caution needs to be exercised. First, the known-groups that were used might not necessarily have been the most indicative for assessing the suitability of EQ-5D-5L for measuring the HRQoL of PwD. It is noted that the authors in the included papers assessed known-group validity based on statistical significance and not on whether the expected differences between groups were clinically relevant despite the latter being recommended by the COSMIN guidelines [48]. In assessing known-group differences between the intervention and treatment groups, non-significant differences could have been the result of an “ineffective” intervention or other factors rather than the psychometric properties of the instrument per se. In the two studies assessing known-group validity across those with and without dementia, one did not find a significant difference and the other found an outcome in the wrong direction, and this may be impacted by under-diagnosis or diagnosis at later disease stages. Similarly, failure of an instrument to detect responsiveness which is change over time may be due to the intervention (and the sample size) rather than the ability of the instrument to detect change; we could not disentangle these in the evidence provided. From the published sources, it was not always clear whether a change was expected with respect to a global rate of change or as assessed by clinicians. From the mixed evidence reported in this paper, there was reassurance that EQ-5D-5L was likely to capture known-group validity and had convergent validity with other measures commonly used in PwD. However, concerns were raised around responsiveness, inter-rater reliability and acceptability and feasibility. Whilst inter-rater reliability and acceptability and feasibility may be an issue only for self-report for PwD and may be equally applicable to other measures where self-reported by PwD, further evidence on this (and head-to-head comparisons of measures) would be beneficial. We recommend that additional analyses are required on secondary datasets to be able to answer some of these questions more accurately.

The review highlighted that as the severity of the condition increased, PwD were less likely to be able to self-complete EQ-5D-5L (or measures in general) because of fatigue, cognitive or functional impairment. It was not possible to determine from the review, the suitability of EQ-5D-5L across different severity levels and other co-morbidities despite this being of crucial importance. It is recommended that more detailed analyses required to make clear recommendations around the suitability of EQ-5D-5L across these variables. This warrants more detailed analyses on secondary datasets that allow for more head-to-head comparisons of different generic and condition-specific PBMs.

Self-completion is not always feasible for several populations including children, those at the end of life, those with several cognitive impairment and PwD at a later stage of disease. Given that a proportion of the population with dementia are unable to self-complete HRQoL, a viable option is for the measures to be completed by proxies. In this review, there was clear evidence of absence of inter-rater reliability of EQ-5D-5L. This finding in dementia is supported by a large literature on this issue [49–54]. In general, PwD themselves tend to provide more optimistic reports of their own HRQoL than their proxies, and there was some evidence that this difference became more pronounced at the more severe stages of disease [55]. The proxies should be a person who knows the PwD and is involved in their care, for example informal carers such as family members and friends [6]; however this closeness in relationship may be contributing to the disparity in reports via projection bias of proxy/caregiver burden. In addition, the wider literature shows that factors such as the relationship of the proxy, and specific characteristics of the proxy themselves can impact proxy assessments of HRQoL [50], as well as more pragmatic aspects such as the perspective the proxy is told to adopt when completing the measure [6, 52], and mode of administration (i.e., telephone, postal or interview) [56]. While the lack of inter-reliability is likely to be equally relevant for other measures, the issue of proxy reporting remains pertinent for EQ-5D-5L as it is the recommended measure for use in economic evaluation. Despite the known differences between self and proxy reports, there is no clear guidance on how to interpret these differences, and which HRQoL-reports to use to generate QALYs. A recent paper made an attempt to do this using psychometric techniques [57]. More research is warranted to contribute to the debate on how to interpret the differences between self-report and proxy-reports that can be more easily reflected in an economic evaluation and may provide a solution when self-report is only possible for a sub-group of the study population.

This review has not been able to throw any light on the comparison of EQ-5D-3L and EQ-5D-5L. One of the motivations for developing the latter measure was to overcome some issues related to EQ-5D-3L such as ceiling and floor effects due to the crude response levels. Li et al. [7] reported that in a trial comparing DEMQOL-U and EQ-5D-3L higher ceiling effects were observed for EQ-5D-3L [8]. Similar findings on high ceiling effects were observed in several studies [50, 52, 58]. We are unable to draw any conclusion on the presence of ceiling and floor effects in EQ-5D-5L in PwD as one paper explicitly reports that no ceiling or floor effects exists while two report evidence of ceiling effects. A more recent paper not included in the review comparing EQ-5D-3L and EQ-5D-5L in PwD suggests that the ceiling effects are 17% lower in the latter compared with the former [59].

## Conclusions

This review based on 20 papers from 14 different studies has reported the following psychometric properties (overall assessment of psychometric property) of EQ-5D-5L with PwD: known-group difference (good), convergent validity (good), responsiveness (inconclusive), reliability (poor), and acceptability and feasibility (moderate). We were unable to assess floor and ceiling effects and there was very limited evidence on content validity. Concerns were raised around the absence of inter-rater reliability and the inability to self-report which have implications for use of utilities generated for economic evaluation. The evidence must be interpreted with caution as the number of studies is limited, and the nature of the studies can mean that evidence of a psychometric property may not be demonstrated due to the specific characteristics of the particular studies rather than a weakness of the EQ-5D-5L.

## Supplementary Information

**Additional file 1**. **Table S1**: Known-group validity (7 studies). **Table S2**: Convergent validity (9 studies). **Table S3**: Reliability (7 studies). **Table S4** Quality assessment of included papers adapted from the GRADE assessment tool.

## Data Availability

The tables supporting the conclusions of this article are included within the article and its additional files.

## Abbreviations

Actifcare: Access to timely formal care cohort
AQOL: Assessment of Quality of Life
CDR: Clinical Dementia Rating
CSDD: Cornell Scale for Depression in Dementia
DEMQOL: Dementia Quality of Life
DEMQOL-U: Dementia Quality of Life-Utility measure
EPIC trial: Enhancing person centred care in care homes
EQ-5D: EuroQoL-5 dimensions
EQ-5D-3L: EuroQoL-5 dimensions 3-level
EQ-5D-5L: EuroQoL-5 dimensions 5-level
EQ-VAS: EuroQoL-visual analogue scale
EQ-VT: EuroQoL-valuation techniques
FAST: Functional Assessment Screening Tool
HRQoL: Health-related quality of life
HUI: Health Utility Index
ICECAP-O: ICEpop CAPability measure for Older people
MBI: Modified Barthel Index
MMSE: Mini-mental state examination
NICE: National Institute for Health and Care Excellence
PAS-Cog: Cognitive Impairment Scale of the Psychogeriatric Assessment Scales
PBM: Preference-based measure
PwD: People with dementia
QALY: Quality-adjusted life year
QWB: Quality of well-being
QoL-AD: Quality of Life-Alzheimer Disease
QoL-AD NH: Quality of Life-Alzheimer Disease Nursing home version
QUALID: Quality of Life in late-stage dementia
TTO: Time-trade-off
VAS: Visual analogue scale
DCM WIB: Dementia care mapping well/illbeing (score)
