# Supplementary material for: Assessing the psychometric performance of EQ-5D-5L in dementia: a systematic review
Source: Health Qual Life Outcomes. 2022 Sep 28;20:139. doi: 10.1186/s12955-022-02036-3 (PMC9520934; doi:10.1186/s12955-022-02036-3)
Supplement: Supplementary file 1 — Additional file 1. Table S1: Known-group validity (7 studies). Table S2: Convergent validity (9 studies). Table S3: Reliability (7 studies). Table S4 Quality assessment of included papers adapted from the GRADE assessment tool. [file 12955_2022_2036_MOESM1_ESM.docx]

Supplementary materials

Table S1: Known-group validity (7 studies)

| **Study reference (author, year)** | **Index or dimensions or both assessed** | **Groups defined as** | **Mean differences across groups in direction consistent with clinical expectation** | **Difference between groups statistically significant** | **Details if applicable** |
| --- | --- | --- | --- | --- | --- |
| Easton, 2018 (14) | Both | By cognition impairment via PAS-Cog score: Mild (0-9), moderate (10-15) and severe (16-21)  By functional impairment in terms of dependence via MBI: no (>100), slight (91-99), moderate (61-90), severe (21-60), total dependence (0-20).  By dementia status: with a diagnosis vs. without a diagnosis. | Dimensions: Yes  Index: Yes for cognitive and functional impairment and No for dementia diagnosis | Yes | Dimensions: differences as expected - Yes- for physical function severity groups (from both self-report and proxy). No - for cognitive impairment defined groups (no strong relationship for proxy report and inverse relationship for self-report).  Mean EQ-5D-5L scores were higher for residents with a diagnosis of dementia compared to those without. |
| Handels, 2018 (7) | Index | Unmet need: no unmet need, 1 or 2 unmet needs, and 3 or more unmet needs | Yes | Yes. Fewer were significant for proxy compared with self-report. | Albeit significant, the differences for self-report were small. Similar pattern for the proxy ratings (and the carer self-report) with fewer of the differences being significant |
| Ratcliffe, 2017 (23) | Index | Self-reported and proxy EQ-5D-5L scores according to: a. cognitive impairment measured by MMSE score: Mild ( >20), Moderate (10–20), Severe (<10); b. Depression measured by CSDD score: Non-case (<11), Probable (11–17), Definite ( >17); c. Self-care measured by MBI dependence score: Independence (100), Slight (MBI score 91–99), Moderate (61–90), Severe (21–60), Total dependence (0–20); d. Pain measured by PainAd score: No pain (0), Mild (1–3), Moderate (4–6), Severe (7–10) | Mixed but overall yes | Mixed but overall yes | All effect sizes were below 0.3 indicating small to moderate overall effect. Differences were significant at baseline for self-reported EQ-5D-5L across all known-groups; significant for all known groups except cognitive impairment.  Differences were non- significant at 4 weeks for self-reported EQ-5D-5L across all known-groups; significant across depression and self-care for proxy completed and non-significant for cognitive impairment and pain |
| Sopina, 2019 (25) | Both | Facilitated family case conferencing (similar to care planning) versus with usual care | Yes | No |  |
| Toh, 2020 (16) | Both | Differing health status based on levels of physical function and communication ability. RAF - Resident Assessment Form Categories - 1) cognitively and physically independent, Category 2) semi-ambulant, Category 3) wheelchair or bedbound, Category 4) highly dependent. | Yes | Yes | Residents with the ability to communicate effectively (P < 0.01) and residents who belonged to RAF categories of 2 and 3 (P < 0.01) had higher EQ-5D-5L index scores than those who were unable to communicate effectively and those of RAF category 4 respectively. All EQ-5D-5L domain scores were better in residents with the ability to communicate and better physical function. The median severity of health problems in the mobility domain was 3 (moderate problems) and 4 (severe problems) for residents with and without communication ability, respectively (p < 0.01); the median pain/discomfort level was 1 (no pain/discomfort) and 2 (slight pain/discomfort) for residents in RAF category 2/3 and 4, respectively (p < 0.01). |
| Umegaki, 2020 (17) | Both | People with and without sarcopenia | Yes | Yes | Proxy-rated utility values were significantly lower in sarcopenia participants; proxy-rated mobility and pain/discomfort domains and both self and proxy-rated anxiety/depression domains were significantly lower in those with sarcopenia; A significant negative association in those with sarcopenia with both self- (p=0.036) and proxy-rated (p=0.002) EQ-5D-5L utility values independent of potential confounding factors. |
| van de Rijt, 2020 (24) | Index | People with and without dementia | Yes | No | EQ5D index score in dementia group: 0.075 (0.279) and non-dementia group: 0.199 (0.344), p = 0.060 |

Notes: CSDD- The Cornell Scale for Depression in Dementia, MBI – Modified Barthel Index, MMSE - Mini-Mental State Examination, PainAd - Pain Assessment in Advanced Dementia Scale, PAS-Cog – Cognitive Impairment Scale of the Psychogeriatric Assessment Scale, RAF – Resident Assessment Form.

Table S2: Convergent validity (9 studies)

| **Study reference (author, year)** | **Other HRQoL measures examined for correlation** | **Significant correlations** | **Regression analysis undertaken** | **Details of analysis** | **Regression analysis shows significant relationship yes/no** |
| --- | --- | --- | --- | --- | --- |
| Easton, 2018 (14) | DEMQoL-U and DEMQoL-proxy-U. | Yes - EQ-5D-5L and DEMQOL-U; EQ-5D-5L utilities and DEMQOL-U. | No |  |  |
| Griffiths, 2020 (11) | QUALID, DEMQoL-proxy, QoL-AD nursing home | Yes - EQ-5D-5L self report significantly correlated with QUALID staff proxy, QUALID relative proxy, QoL-AD self- report , DEMQoL and DEMQoL relative proxy (0.39 <0.01). EQ-5D-5L staff proxy significantly correlated with QUALID staff proxy, QUALID relative proxy, DEMQoL staff proxy. EQ-5D-5L relative proxy significantly correlated with QUALID, QUALID relative proxy, QoL-AD and DEMQoL relative proxy. | No |  |  |
| Janssen, 2018 (8) | ICECAP-O | Yes - positive significant correlation between ICECAP-O and EQ-5D- 5L utilities at baseline | No (not for EQ-5D-5L) |  |  |
| Martin 2019 (12) | DEMQoL-Proxy-U, QOL-AD-NH, QUALID | Yes - resident-reported EQ-5D-5L and formal- carer–completed QUALID. | Yes - Spearman's rank (Bonferroni adjusted) regression analysis | Panel data regression models with random effects to measure association between HRQoL measures (QUALID and QOL-AD-NH) | Significant relationship between both EQ-5D-5L-proxy utility scores and the FAST and CDR measures. |
| Perry-Duxbury, 2020 (9) | ICECAP-O in the informal caregiver | Yes - ICECAP-O tariff significantly associated with EQ-5D-5L utility tariff score | Yes |  | Yes - Significant association between EQ-5D-5L and ICECAP-O |
| Ratcliffe, 2017 (23) | DEMQOL-Proxy U | Yes - Proxy completed EQ-5D-5L and DEMQOL-Proxy U; Yes - EQ-5D-5L and MMSE | No |  |  |
| Rombach, 2020 (10) | QoL-AD scores and EQ-5D-5L utilities. In supplementary materials also reported for QOL-AD items and EQ-5D-5L dimensions. | Yes - between similar dimensions in QOL | Yes |  |  |
| Sopina, 2019 (25) | QUALID | Yes - significant correlations between QUALID and EQ-5D-5L. | Yes | Linear regressions to investigate the relationship between changes in EQ-5D-5L and QUALID over 3-month intervals, and controlled for age, gender, as well as the EQ-5D-5L and QUALID scores at the start of each interval being assessed | Yes |
| Toh, 2020 (16) | Domains of EQ-5D-5L and DCM WIB | Yes - significant correlation between EQ-5D-5L index and the DCM Well/Ill being value | No |  |  |

Notes: CDR: Clinical Dementia Rating, DCM WIB - Dementia care Mapping Well/Ill being (score), CDR - DEMQOL- Dementia Quality of Life, DEMQOL-U Dementia Quality of Life Utility measure, FAST – Functional Assessment Screening Tool, ICECAP-O - ICEpop CAPability measure for Older people, QoL-AD - Quality of Life – Alzheimer Disease, ), QoL- AD NH - Quality of Life – Alzheimer Disease Nursing Home version, QUALID - Quality of Life in late-stage dementia

Table S3: Reliability (7 studies)

| **Study reference (author, year)** | **Index or dimensions or both assessed** | **Analysis** | **Reliability observed** | **Relevant information** |
| --- | --- | --- | --- | --- |
| Griffiths, 2020 (11) | Both | Inter-rater reliability by self, proxy (relatives or friends or care staff); weighted Cohen’s Kappa statistic | No | Dimensions: Fair agreement between staff proxy and relative/friend proxy ratings; poor agreement between the 377 cases of staff proxy and resident ratings. Poor agreement: relative/friend proxies and residents (n = 80). EQ-5D-5L index: Low agreement between resident and relative/friend ratings. Very low agreement between resident and staff proxy ratings, not statistically different. Low agreement between the staff and relative/friend proxy ratings. |
| Handels, 2018 (7) | Index | Inter-rater reliability by self and proxy (informal caregiver); paired t-tests | No | Significant differences for all self- and proxy ratings. |
| Martin 2019 (12) | Index | Inter-rater reliability by self, proxy (formal and informal carers) assessed by spearman rank-order correlation and Bland Altman plots | Overall No. (Yes for informal carer and formal carer proxy completed; No for informal carer proxy and PwD self- completed; No for formal carer proxy and PwD self-completed) | Correlation between formal-carer EQ-5D-5L-proxy and resident-reported EQ-5D-5L utility scores was weaker (rho = 0.264) than that between informal-carer EQ-5D-5L-proxy and resident-completed EQ-5D-5L utility scores (0.354). On average, EQ-5D-5L-proxy scores were lower than the resident- completed EQ-5D-5L, with the greatest discrepancies between utility scores at lower utility levels. |
| Sopina, 2019 (25) | Both | Inter-rater reliability; self and proxy (nurse). Intra- class correlation coefficients for residential facilities and two-way mixed effects model regression. | No | Analyses reported the intra-class correlations of different residential facilities to be very low or no correlations between individuals, suggesting poor reliability between raters, or a potential lack of variability among raters, as evidenced by little variation in the data. |
| Sopina, 2017 (18) | Index | Inter-rater reliability; self and proxy (main caregiver) ; Probability of being cost-effective - sensitivity analysis | No | Probability that the intervention is cost-effective in comparison with the control is higher when using participant assessments than the caregiver assessment. |
| Umegaki, 2020 (17) | Both | Correlation analysis between self and proxy (main caregiver) | No | The correlation coefficient between self- and proxy- rated values was 0.427, and the correlation coefficient between utility and VAS scores was 0.431 for self- ratings and 0.434 for proxy rating |
| Usman, 2019 (22) | Both | Inter-rater reliability staff proxy and self-complete at three time points. Weighted kappa statistics and intra-class correlation coefficients (ICCs) adjusted for clustering at the care home level were used to measure agreement between resident and staff proxies for each time point | No | Fair agreement for the mobility dimension of EQ-5D-5L and slight for all other dimensions; EQ-5D-5L index - fair agreement. Staff proxy ratings consistently differ from those of residents, for residents with or without cognitive impairment. |

Table S4 Quality assessment of included papers adapted from the GRADE assessment tool

|  | ***Population*** | ***Sample*** | | | ***Outcome assessment*** | | ***Analysis*** | | ***Data*** | | ***Limitations*** | **Quality score** |
| --- | --- | --- | --- | --- | --- | --- | --- | --- | --- | --- | --- | --- |
|  | **1.** | **2.** | **3.** | **4.** | **5.** | **6.** | **7.** | **8.** | **9.** | **10.** | **11.** |  |
| **Easton 2018** | Y | High | Y | Y | U | Y | Y | Y | N | U | N | Medium |
| **Engel 2020** | Y | Low | Y | Y | N/A | Y | Y | N/A | N/A | N/A | N/A | Undetermined |
| **Griffiths 2020** | Y | High | Y | Y | U | Y | Y | Y | N | Y | N | High |
| **Handels 2018** | Y | High | Y | U | N/A | Y | Y | Y | N | Y | Y | High |
| **Harrison 2018** | Y | High | Y | N | U | N | Y | Y | N | N | N | Medium |
| **Hurley 2020** | Y | Low | N | U | N/A | Y | Y | Y | U | N/A | N | Low |
| **Janssen 2018** | Y | High | Y | Y | N/A | Y | Y | Y | N | N | Y | High |
| **Jurkeviciute 2019** | Y | Low | Y | U | N | Y | Y | Y | N | N/A | N | Low |
| **Maidment 2020** | Y | Low | Y | Y | N | Y | N | Y | Y | N/A | Y | Medium |
| **Martin 2019** | Y | High | Y | N | U | Y | Y | Y | N | Y | Y | High |
| **Meads 2020** | Y | High | Y | N | U | N | Y | Y | N | Y | N | Medium |
| **Perry-Duxbury 2020** | Y | High | Y | U | N/A | Y | Y | Y | N | N | Y | Medium |
| **Ratcliffe 2017** | Y | Medium | Y | Y | N | Y | Y | Y | N | N | N | Medium |
| **Rombach 2020** | Y | High | N/A | U | N/A | Y | Y | Y | N | N | N | Medium |
| **Sopina 2019** | Y | Medium | Y | N | Y | Y | Y | Y | U | N/A | Y | Medium |
| **Sopina 2017** | Y | Medium | Y | Y | U | N | Y | Y | N | Y | N | Medium |
| **Toh 2020** | Y | Medium | Y | Y | N/A | Y | Y | Y | N | Y | N | Medium |
| **Umegaki 2020** | Y | Low | N | Y | N/A | N | Y | Y | U | N | N | Low |
| **Usman 2019** | Y | Medium | N | U | U | Y | Y | Y | U | N | N | Medium |
| **van de Rijt 2020** | Y | Low | Y | Y | N/A | N | Y | Y | U | N/A | N | Medium |

^a^ U – Undetermined Questions: 1. Is the study population clearly defined? 2. What is the magnitude of the sample size? High >300 = 2, intermediate 100-300 = 1, low >100 = 0 3. Are sample groups clearly defined? 4. Are >80% of participants enrolled in the study included in the analysis? 5. Was there blinding of outcome assessments? 6. Are administration details clearly indicated? 7. Are details of analyses 8. Are relationships measured via statistical methods i.e., correlation, p value, 95% CI provided? 9. Is the data complete? 10. Have the authors provided thresholds for missing data? 11. Was selection bias discussed?

Each question was scored 1 if Y, 0 if no or undetermined for all questions except question 2 where sample >300; 100- 300; <100 score 2, 1 and 0 respectively. The score was then calculated for the 11 questions = 12 points, Can define >9 = high, 5-8 = medium, <5 = low?
